# Previously unidentified Indonesian Throughflow pathways and freshening in the Indian Ocean during recent decades

**DOI:** 10.1038/s41598-019-43841-z

**Published:** 2019-05-14

**Authors:** Salvienty Makarim, Janet Sprintall, Zhiyu Liu, Weidong Yu, Agus Santoso, Xiao-Hai Yan, R. Dwi Susanto

**Affiliations:** 1 State Key Laboratory of Marine Environmental Science, and Department of Physical Oceanography, College of Ocean and Earth Sciences, Xiamen University, Xiamen, China; 2 Agency for Marine and Fisheries Research and Development, Ministry of Marine Affairs and Fisheries, Jakarta, Indonesia; 3 Scripps Institution of Oceanography, University of California San Diego, La Jolla, CA USA; 4 First Institute of Oceanography, SOA, Qingdao, China; 5 Climate Change Research Centre and ARC Centre of Excellence for Climate Extremes, University of New South Wales, Sydney, Australia; 6 Centre for Southern Hemisphere Oceans Research, CSIRO Oceans and Atmosphere, Hobart, Australia; 7 College of Earth, Ocean and Environment, University of Delaware, Newark, DE USA; 8 Department of Atmospheric and Oceanic Science, University of Maryland, College Park, MD USA; 9 Faculty of Earth Sciences and Technology, Bandung Institute of Technology, Bandung, Indonesia

**Keywords:** Attribution, Physical oceanography

## Abstract

The Earth has experienced a global surface warming slowdown (GSWS) or so-called “global warming hiatus” since the end of the 20^th^ century. The GSWS was marked by a La Niña-like decadal cooling in the Pacific Ocean that subsequently generated an increase in the transfer of Pacific waters into the Indian Ocean via the Indonesian Throughflow (ITF). How the Pacific water spreads through the interior of the Indian Ocean and the impact of these decadal ITF transport changes on the Indian Ocean water mass transformation and circulation remain largely unknown. Here, we analyze the thermohaline structures and current systems at different depths in the Indian Ocean prior to and during the GSWS period. Our study shows that the GSWS involved extensive changes to the Indo-Pacific ocean teleconnection system, characterized by subsurface warming and freshening in the Indian Ocean. A hitherto unknown Indian Ocean pathway of the ITF was discovered off Sumatra associated with prolonged northwestward flow within the South Java Current. Our analysis uncovers a direct linkage of enhanced ITF waters with the Agulhas Current in the Mozambique Channel from thermocline depths down to intermediate depths, that freshened the Indian Ocean. These changes in the Indian Ocean circulation and water mass characteristics impact climate variability through changing the sea surface temperature (SST) and precipitation patterns that can subsequently affect regional economies.

## Introduction

Despite an unabated increase in anthropogenic greenhouse gas emission, a decadal period of global surface warming slowdown (GSWS) has occurred since the end of the 20^th^ century. The GSWS was characterized by notable changes in global climate and ocean circulation, marked by a surface cooling over the eastern tropical Pacific that resembled a La Niña pattern^1^. Correspondingly, an enhancement of the Pacific trade winds^2^ increased heat uptake from the atmosphere into the ocean, leading to an increase in heat stored in the tropical Pacific^2^. This excess heat was transmitted into the Indian Ocean via the Indonesian Throughflow (ITF)^3^ as part of the energy redistribution process across oceanic basins^4^. The oceanic channel of the Indo-Pacific plays an important role in connecting the interannual oceanic variations via the propagation of equatorial waves^5^ associated with both the Indian Ocean Dipole (IOD) and the El Niño–Southern Oscillation (ENSO)^6^. Yet the implications of the GSWS on the Indian Ocean circulation remains poorly understood and warrants further investigation since the Indian Ocean plays an important role in regulating global and regional climate. In particular, the IOD climate mode exerts significant impact over Indian Ocean-rim countries and affects the climate of remote regions^7,8^. This study aims to paint a clearer picture of how the Indian Ocean circulation changed during the GSWS in association with the increase of the ITF transport.

As part of the global thermohaline circulation^9^, the ITF influences the heat and freshwater budgets of both the Indian and Pacific Oceans^10^ and is essential in maintaining the state of the global climate^11^. The ITF is dominated by low salinity North Pacific water masses in the upper thermocline and more saline South Pacific water masses in the lower thermocline^12^ and deeper layers. These Pacific waters primarily enter the Indonesian Seas through Makassar Strait with a smaller contribution entering through Lifamatola Passage, the South China Sea (SCS) and Karimata Strait^13–16^. The contribution of buoyant South China Sea (SCS) water may impede the surface layer flow through Makassar Strait during El Niño^17^ and during the rainy winter monsoon^13,18,19^. The majority of the Pacific waters exit into the Indian Ocean via Lombok, Ombai and Timor Straits^20^ and a small portion via the Sunda Strait during boreal summer^16^. The complex and unique bathymetry of the narrow passages along the ITF pathways in the Indonesian Seas generate strong tidal mixing^20,21,22^, altering the Pacific water properties. However, important questions remain as to how the ITF spreads in the Indian Ocean after flowing out from the Indonesian Seas and consequently impacts the Indian Ocean circulation.

The ITF spreading in the Indian Ocean is mainly associated with the South Equatorial Current (SEC)^23^. The ITF has two cores in the Indian Ocean: a salinity minimum in the upper 300 m (Indonesian Upper water; IUW)^24^ and a deeper core of low salinity, and high silica (Indonesian Intermediate Water; IIW)^25^. How these two ITF cores circulate and interact with the water masses and the current system in the Indian Ocean is poorly understood. On interannual time scales, the ITF transport is thought to be weaker during an El Niño^26,27^ and stronger in a La Niña^28,29^. However, this interplay is influenced by the IOD^30^ through Kelvin and Rossby wave propagation in the Indian Ocean^30–32^ which modifies the depth and velocity of the ITF cores through heat exchanges^20^. Eddy activity in the South East Tropical Indian Ocean (SETIO)^33^ can also influence the ITF streams causing decadal subsurface cooling in the Indian Ocean^34^. Further downstream, the linkage of the ITF and the Agulhas Current remains obscure.

To unravel how the ITF spreads from its exit passages into the interior of the Indian Ocean, we examine salinity structures along isopycnal surfaces extending from the Lombok, Ombai and Timor exit passages^20^ using ORAS4 ocean reanalysis. Temperature-salinity diagrams reveal the spatial evolution of the properties along the ITF pathways. This allows a calculation of the “ITF water fraction” in these regions (ref.^35^, Equation-2) assuming that the ITF water represents an intrusion of water in the Indian Ocean that spreads mainly along isopycnal surfaces σ_θ_ (see Methods). Changes in the ITF pathways in the upper layer of the Indian Ocean can also affect the thickness of the salinity-stratified barrier layer (BLT) and so lead to changes in air-sea interactions^20^. The impact of a stronger ITF during the GSWS period and subsequent changes in the ITF pathways on the Indian Ocean current system and barrier layer are discussed.

## Pathways and Water Mass Characteristics of the ITF During Recent Decades

**Figure 1:**
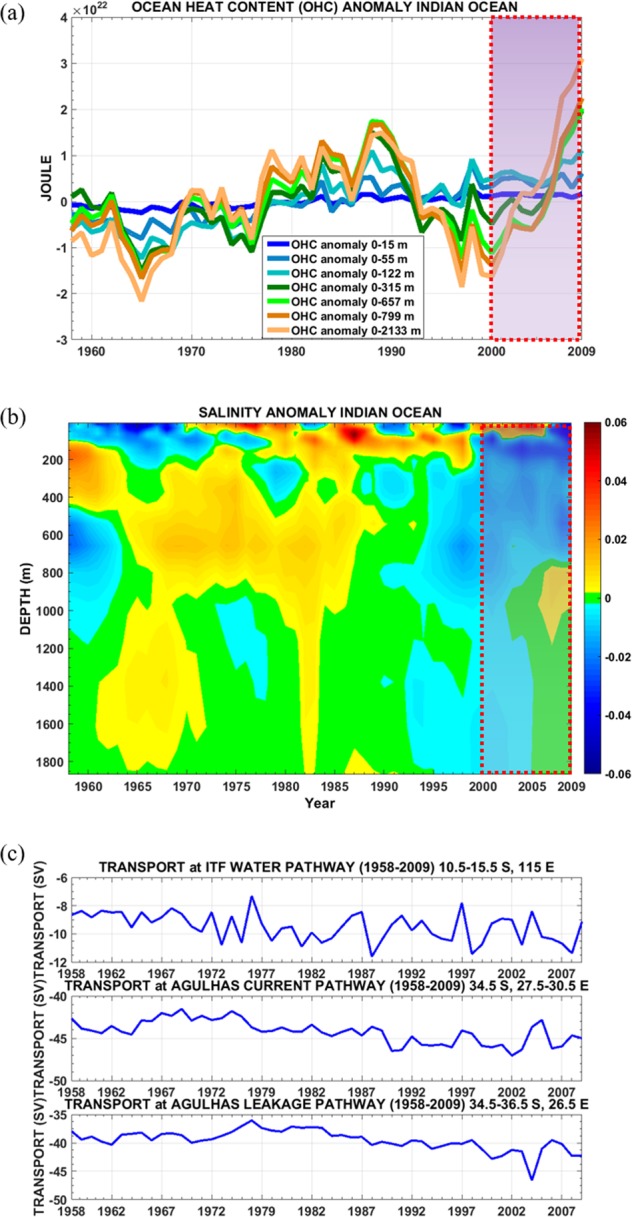
Temporal evolution of ocean heat content (OHC) anomaly and salinity anomaly in the Indian Ocean. (**a**) OHC anomaly (unit: Joule) showing subsurface warming since 2000 (red box) in the Indian Ocean over various depth ranges indicated in the inset. (**b**) Salinity anomaly (unit: psu) indicating subsurface lower salinity from 1997–2009. The anomalies are calculated from the ORAS4 dataset as a deviation from the long-term mean (averaged over 20.5°N–40.5°S, 20.5–120.5°E) from 1958 to 2009. (**c**) Transport variability (unit: Sv) of the ITF water (upper panel), Agulhas Current (middle panel) and Agulhas Leakage (lower panel).

The Indian Ocean exhibited rapid subsurface warming since early 2000 (Fig. 1a) that is consistent with the heat content changes in the Indian Ocean^36^ along with a concomitant freshening (Fig. 1b). This occurred during the period of strengthening ITF transport^3^ (Fig. 1b, red box, and Fig. 1c) in association with the global warming hiatus. The transport changes can be seen in the ITF pathway, the Agulhas Current and Agulhas Leakage pathways during 2000–2009 (Fig. 1c). Hereafter we refer to 2000–2009 as the GSWS period and 1958–1999 as the global surface warming (GSW) period, consistent with trends in global mean surface air temperature^2^. Total transport of the ITF water (10.5–15.5°S, 115.5°E), Agulhas Current (34.5°S, 27.5–30.5°E), and Agulhas Leakage (34.5–36.5°S, 26.5°E) during the GSWS period were stronger than in the GSW period (Table S1). Thus, stronger ITF translates to enhanced transport of the Agulhas Current and Agulhas Leakage leading to increased transport from the Indian Ocean to the South Atlantic. Our results suggest that the ITF water from the exit passages (10.5–15.5°S, 115°E) can reach the Agulhas Current region within 12 years and reach the Agulhas Leakage another 10 years later (Fig. S1). This time scale is consistent with a previous estimate^37^ that the upper layer ITF can reach the Agulhas Leakage in about 20 years.

The ITF transport is more variable than that of the Agulhas Current and Agulhas Leakage (Fig. 1c). Over the period 1958–1975, a negative Interdecadal Pacific Oscillation (IPO) occurred concurrently with the earlier period of global warming hiatus^38^. However, the ITF during this period was on average weaker (above 99% statistical confidence level) compared to the GSWS period (2000–2009).

**Figure 2:**
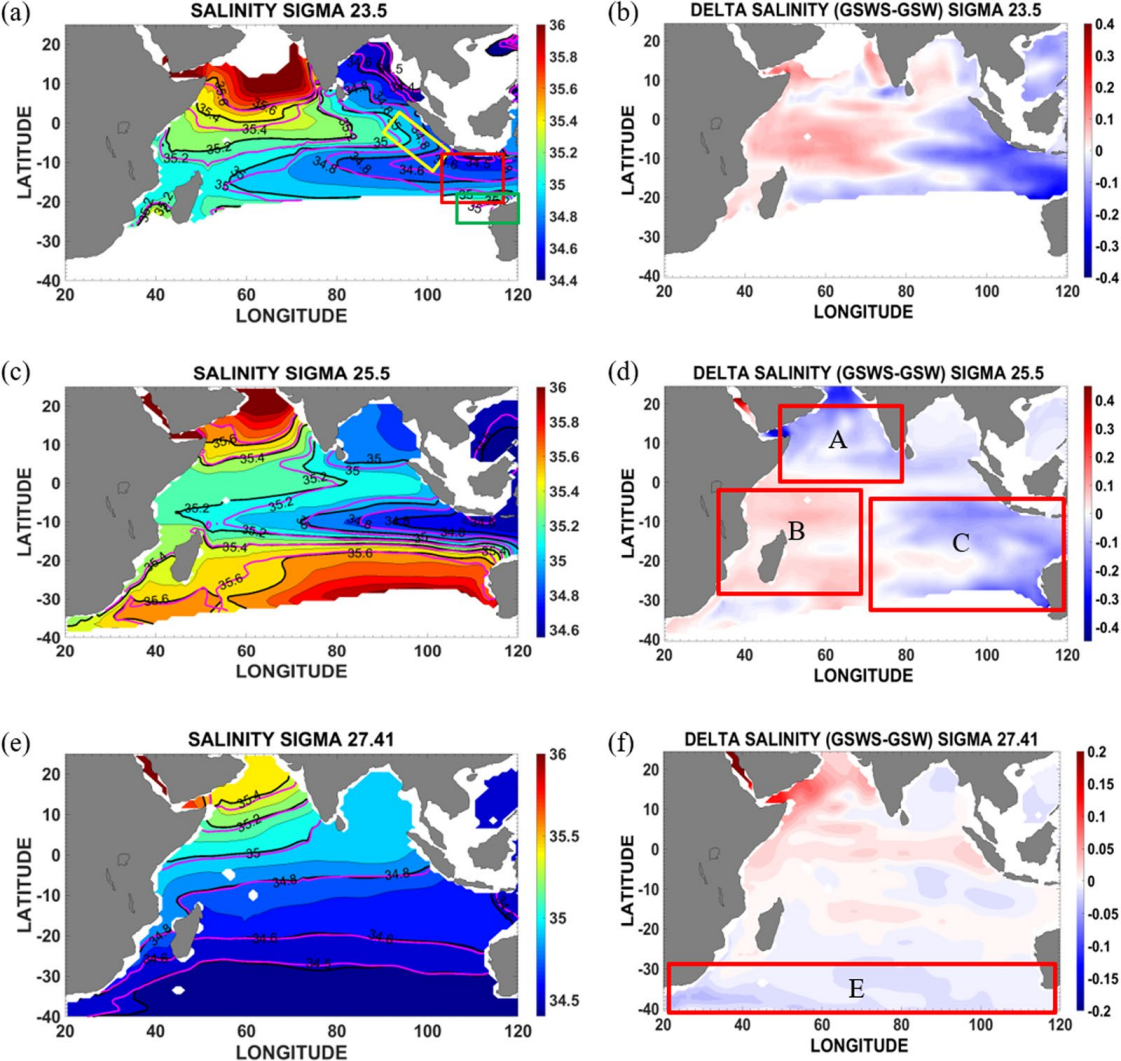
ITF spreading pathways in the Indian Ocean during the Global Surface Warming (GSW) and Global Surface Warming Slowdown (GSWS) periods and contributions to the salinity difference. (**a**) Salinity along σ_23.5_ (mixed layer). Black contours are for the GSW period, pink contours are for GSWS period. Red box is the SETIO eddies location (10.5–15.5°S, 100.5–114.5°E). Green box indicates the deflection of the ITF feeding into the Leeuwin Current and the yellow box the ITF feeding off Sumatra. (**b**) Salinity difference between the GSWS and GSW for σ_23.5_ (mixed layer). (**c**) As in **a** but for σ_25.5_ (thermocline layer). (**d**) As in (**b**) but for σ_25.5_. (**e**) As in (**a**) but for σ_27.41_ (intermediate layer). (**f** ) As in **b** but for σ_27.41._ Box A for Arabian Sea, Box B for Western Indian Ocean, Box C for Eastern Indian Ocean, Box D for Sub-Tropical Mode Water (STMW), and Box E for region of 25–40°S toward the Southern Ocean sector.

The intrusion of the ITF into the Indian Ocean near the ITF exit passages is stronger in the GSWS period than in the GSW period (Fig. 2). This is evident particularly on the σ_25.5_ isopycnal (Fig. 2c; comparing pink and black salinity contours): the low salinity minimum (34.8 psu) extends farther west in the GSWS period. Within the SEC upper thermocline, the ITF waters show a salinity range of 35–35.2 psu (ref.^37^), flowing westward and south toward the Mozambique Channel, and also north toward the Arabian Sea (Fig. 2c)^37,39^. At intermediate depths, the ITF crosses the Indian Ocean toward the east coast of Madagascar^25^ and into the Mozambique Channel (Fig. 2e). Another direct ITF route in the Indian Ocean occurs along the Western Australian coast, where the ITF feeds the Leeuwin Current before deflecting to the east coast of Madagascar (Fig. 2a,c,e)^40^.

Here we point out new and little verified pathways of the ITF water within the Indian Ocean on decadal time scales which became more apparent during the GSWS. ITF water is discernible off Sumatra in the mixed layer (Fig. 2a). During the GSWS, the ITF advection was stronger in the Indian Ocean along 10–15°S and fed into the Mozambique Channel (providing a direct linkage of the ITF and the Agulhas Current) down to intermediate depths (Fig. 2c,e) at a salinity of 34.8 psu (Fig. 2e) that is identified as IIW. IIW is a mixture of South Pacific Water and Banda Sea Water^25,41^. The ITF waters associated with the Agulhas Current flow southward as a boundary current to the Agulhas Leakage region (Fig. 2c,e). This pathway was predicted by previous work^37,39^ although to date there has been little observational verification. The recent Argo data during the GSWS also support these results (see Fig. S2a–c).

The water off Sumatra (Fig. 2e, salinity 34.8 psu) is directly linked to the Mozambique Channel that feeds the Agulhas eddies. Water from the Malacca Strait found off Sumatra^42^ mixes with water from Bay of Bengal, as well as mixes with the water from Sunda Strait and the ITF water within the SJC off Sumatra. The pathway suggested by the 34.8 psu salinity track is similar to the Rossby wave pathway from off Sumatra to the Mozambique Channel^43^. Upon reaching the eastern boundary, eastward propagating Kelvin waves propagate poleward along the Sumatra coast and generate Rossby waves that propagate west to the Mozambique Channel, so influencing the Mozambique Channel eddies. The IIW can also be detected off the Western Australian coast mixing with Antarctic Intermediate Water (AAIW) and attaining a salinity value of 34.6 psu (Fig. 2e).

The difference in salinity between the GSWS and GSW period in the mixed layer and thermocline layer resembles an IOD pattern with fresher waters in the east than in the west (Fig. 2b,d). The fresher waters (0.1–0.3 psu) reach Madagascar along the 15–20°S pathway (Fig. 2b). A freshening signal is also seen at thermocline level (0.1–0.3 psu) over the eastern tropical Indian Ocean (Fig. 2d, box C), Arabian Sea (Fig. 2d, box A), and in the formation area of Sub-Tropical Mode Water (STMW) (Fig. 2d, box D), as well as at intermediate depths (0.05–0.1 psu) toward the Southern Ocean sector (Fig. 2f, box E). Thus, the freshening over the Indian Ocean during the GSWS period is not only due to stronger ITF advection but also due to fresher Arabian Sea water (ASW) at thermocline depth, as well as a freshening^44^ and stronger infiltration^45^ of AAIW (Fig. 2b,d,f).

**Figure 3:**
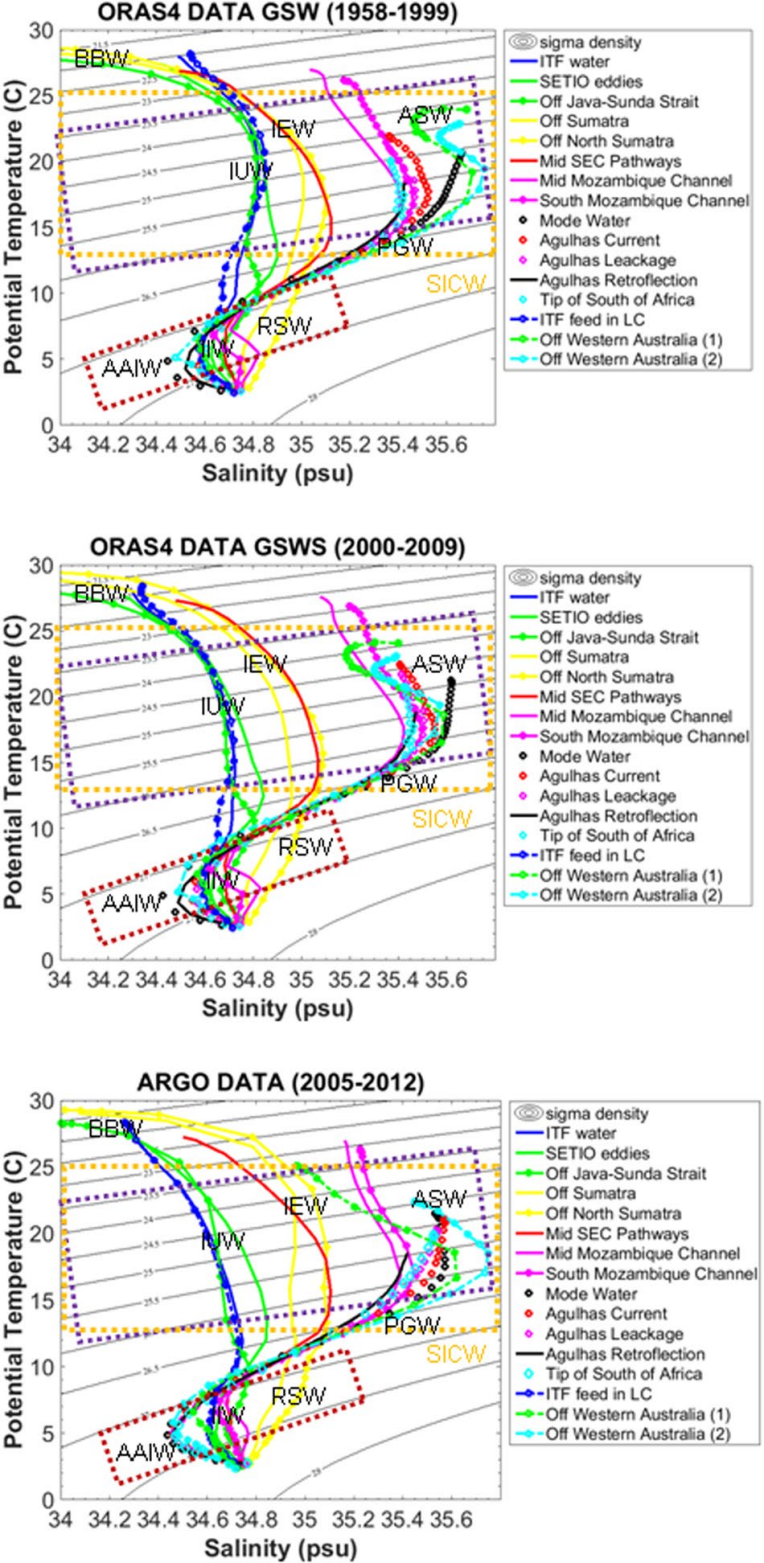
Temperature-salinity (T-S) diagrams in the Indian Ocean. Purple box represents the thermocline layer, brown box the intermediate layers, and yellow box the South Indian Central Water (SICW)^46^. ITF water (blue line, 10.5–15.5°S, 115.5°E), SETIO Eddies (green line, 10.5–15.5°S, 100.5–114.5°E), off Java-Sunda Strait (green circle-line, 6.5–10.5°S, 100.5–107.5°E), off Sumatra (yellow line, 0–6.5°S, 95.5–105.5°E), off North Sumatra (yellow circle-line, 0–3.5°N, 95.5–100.5°E), mid SEC pathways (red line, 10.5–15.5°S, 70.5–90.5°E), mid Mozambique Channel (pink line, 19.5°S, 42.5–43.5°E), South Mozambique Channel (pink circle-line, 22.5°S, 42.5–43.5°E), Mode Water (black diamond, 30.5–34.5°S, 34.5–53.5°E), Agulhas Current (red diamond, 34.5°S, 27.5–30.5°E), Agulhas Leakage (pink diamond, 34.5–36.5°S, 26.5°E), Agulhas Retroflection (black line, 35.5–40.5°S, 20.5–40.5°E), tip of South of Africa (cyan diamond, 34.5–36.5°S, 23.5°E), ITF feed in LC (blue diamond-dash line, 11.5–15.5°S, 115.5–119.5°E), off Western Australia (green diamond-dash line, 22.5–25.5°S, 113.5°E, and cyan diamond-dash line, 26.5°S, 112.5°E). (**a**) T-S diagram from ORAS4 data during the GSWS (2000–2009). (**b**) T-S diagram from ORAS4 data during the GSW (1958–1999) and (**c**), T-S diagram from Argo data (2005–2012). BBW (Bay of Bengal Water), IUW (Indonesian Upper Water), IEW (Indian Equatorial Water), SICW (South Indian Central Water), ASW (Arabian Sea Water), PGW (Persian Gulf Water), RSW (Red Sea Water) and IIW (Indonesian Intermediate Water), (see Method, table watermass list).

The long-term averaged T-S property using Argo and ORAS4 data provide some hints to the spreading of ITF in the Indian Ocean (Fig. 3). In the SETIO eddies region (Fig. 3a–c green line), the T-S property is closer to that from the ITF at the exit region (10.5–15.5°S, 115.5°E) (Fig. 3a–c, blue line). The stratification is likely influenced by the Lombok Strait ITF outflow (Fig. 2 of ref.^20^) rather than that from Timor Passage^23^; the Lombok Strait is a more direct ITF pathway from the Makassar Strait^24,30^. Off Sumatra (Fig. 3a–c, yellow circle-line), the surface layer is influenced by fresh Bay of Bengal Water (BBW)^46^, while the mixed layer (σ_23–23.5_) is influenced by the ITF waters from south of Java (IUW)^46^. In deeper layers (σ_24–27_) there is evidence of Indian Equatorial Water (IEW)^46^, and at σ_27_ there is a penetration of Red Sea Water (RSW)^47^. The T-S property from the Java-Sunda Strait region (Fig. 3a–c, green circle-line) indicates the presence of thermocline ITF water (IUW)^46^ which originates from the ITF water in the SETIO eddies region.

In the south tropical Indian Ocean, the T-S properties of the SEC associated with the ITF pathway (Fig. 3a–c, red line) follow the SETIO eddies properties and have the higher salinity range of the SEC^37^. The T-S properties in the thermocline layer are progressively eroded along the SEC pathway toward the Mozambique Channel (Fig. 3a–c, pink line), presumably due to mixing with the South Indian Central Water (SICW)^37,48^. However, at intermediate depths (σ_θ_ = 27–27.5) the ITF water sourced from the IIW^25,41^ preserves its T-S property (Fig. 3a–c). South of Madagascar near the STMW formation region (Fig. 3a–c, black diamond) and the Agulhas Retroflection (Fig. 3a–c, black line), the deeper layers show AAIW intrusion^46^.

During the GSWS period, the ITF waters at thermocline depths (σ_θ_ = 24–26) were fresher than during the GSW period in many Indian Ocean regions: off Timor (10.5–15.5°S, 115.5°E) (Fig. 3a,b, blue line), the SETIO eddies region (Fig. 3a,b, green line), off Java-Sunda Strait (Fig. 3a,b, green circle-line), and off Sumatra (Fig. 3a, yellow line). Lower salinity is found at intermediate depths (σ_θ_ = 27–27.5) during the GSWS period in the area of the Agulhas Current (Fig. 3b, red line), Agulhas Leakage (Fig. 3b, pink diamond) and at the tip of South of Africa (Fig. 3b, cyan diamond), presumably due to mixing with IIW and AAIW.

The ITF feeds the Leeuwin Current directly and via recirculation within the Eastern Gyral Current^49^ (Fig. 3a–c, blue diamond-dash line) transporting the T-S signature from the ITF inflow region (10.5–15.5°S, 115.5°E) (Fig. 3a–c, blue line). The water farther south off Western Australia (Fig. 3a–c, green diamond-dash line) in the thermocline layer (σ_θ_ = 24.5–26) has higher salinity than the SEC, possibly due to mixing with the Subtropical Water (STW) associated with the South Indian Countercurrent (SICC)^40,50^. At intermediate depth (σ_θ_ = 27–27.5), however, the salinity drops off rapidly, a result of IIW mixing with AAIW. During the GSWS period, this ITF pathway along the Western Australian coast had lower salinity in the thermocline layer (Fig. 3a–c, σ_θ_ = 24.5–26, blue diamond-dash line and green diamond-dash line).

## Mixing of ITF and Agulhas Current in the Mozambique Channel

In the Mozambique Channel, mixing occurs between different water masses: the ITF water advected via the SEC^37,48^ while the Subtropical Surface Water and the Tropical Surface Water form the main source waters within the upper layer^51^. A direct linkage of the Agulhas Current and ITF water in the Mozambique Channel^52^ occurs at thermocline and intermediate depths (Figs 2c,e and 3a–c, pink line, σ_θ_ = 25–27.5) with some mixture of saltier Arabian Sea/Persian Gulf/Red Sea waters along the SEC pathway. In addition, there is a possible penetration from the South East Madagascar Current^52,53^ as a diluted ITF water with SICW^37,48^ flowing to the south of Madagascar and into the southern end of the Mozambique Channel (Fig. 2e). This is plausible particularly since anomalous salinity from the southeast Indian Ocean has been identified to participate in the boundary current of southeast Madagascar^53^.

## Itf Water Subduction and Sjc Northward Deflection to Sumatra

**Figure 4:**
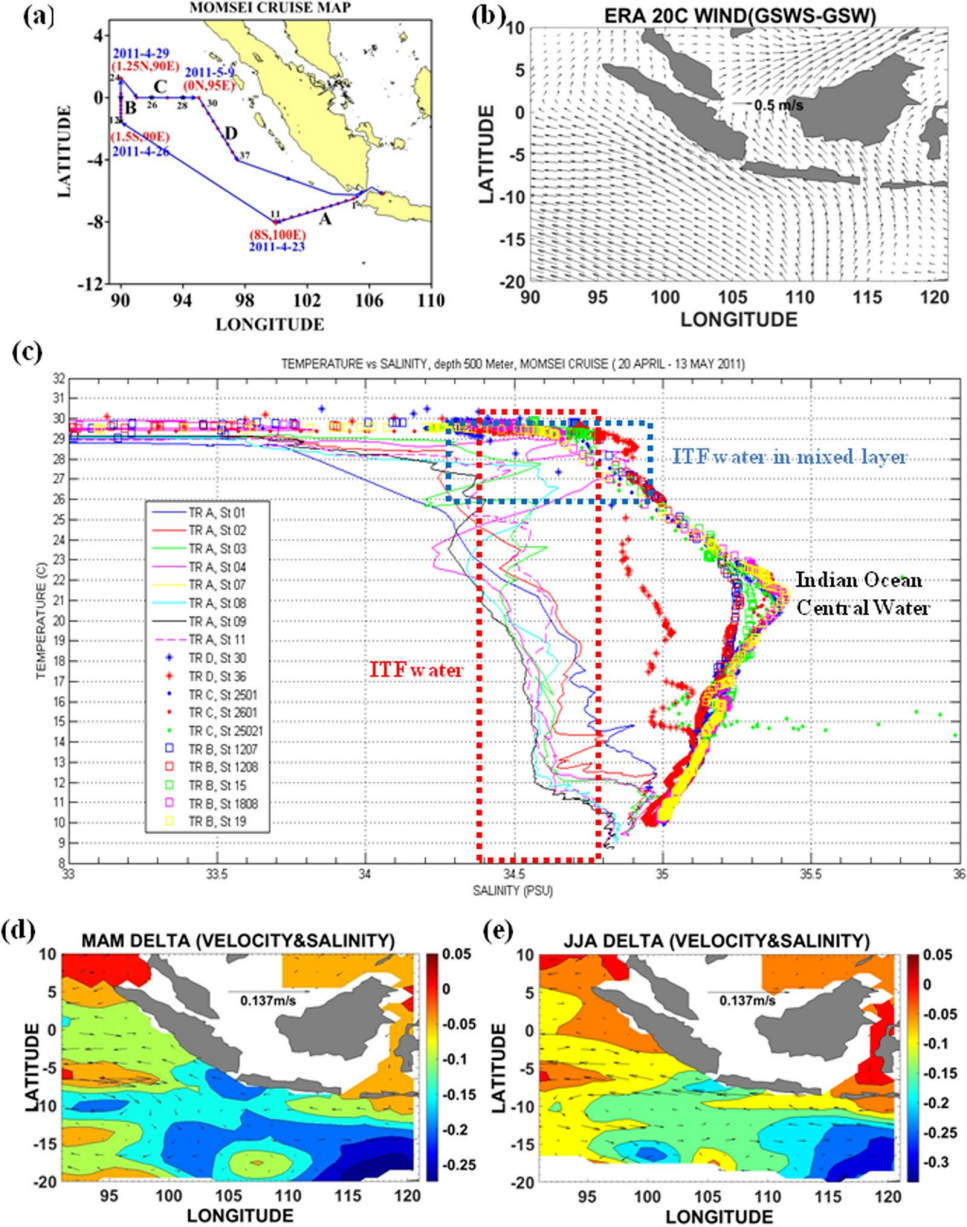
ITF water in the mixed layer off Sumatra observed during the Monsoon Onset Monitoring and Social Ecological Impacts (MOMSEI) cruise in 2011 and the South Java Current northward deflection off Sumatra represented by ORAS4 Delta velocity (GSWS-GSW) and Delta salinity (GSWS-GSW) at average depth of σ_θ_ = 23.5–24.5 layer. (**a**) MOMSEI cruise map. (**b**) The wind pattern difference (GSWS-GSW period) (unit: m/s) from ERA 20 C dataset showing the enhanced easterly wind. (**c**) The T-S diagram from CTD casts during the MOMSEI cruise (20 April–13 May 2011) confirmed the ITF water in the mixed layer off Sumatra (track BCD, blue box) and the ITF water presence up to 500 m depth off Sunda Strait (track A, red box). (**d**) Delta Velocity and Delta Salinity during March–April–May (MAM). (**f**) Delta Velocity and Delta Salinity during June-July-August (JJA).

The ITF presence has been noted previously^54–56^ in upwelled water west of Sumatra during the boreal summer monsoon. Here, we find that the ITF can reach off Sumatra in the upper ocean (Fig. 2a) directly from south of Java. This pathway is more likely during the GSWS period when the SEC is stronger and wider. Analysis of stratification and water characteristics based on the Monsoon Onset Monitoring and Its Social Ecological Impacts (MOMSEI) CTD cruise data confirmed the presence of the ITF source water in the mixed layer off Sumatra in April–May 2011 (Fig. 4c, track BCD, blue box) at 70–90 m depth in the salinity range 34.5–34.8 psu, and in the temperature range 26°–29 °C (Fig. S4). Off Sunda Strait the ITF was found from the surface to 500m depth (Fig. 4c, track A). The SJC is the seasonally reversing coastal current driven by the monsoon wind^57–59^. During GSWS, the SJC was strongly north-westward in March-August (Fig. 4d,e) in response to enhanced southeasterly winds (Fig. 4b) associated with the decadal cooler tropical Pacific^2^. On an advective time scale, the ITF water from South of Java would reach off Sumatra in about 9 months (based on a typical velocity of the order of 10 cm/s; ref.^60^). The ITF water off Sumatra in the mixed layer (Figs 2a, 3a–c, yellow circle, σ_θ_ = 23–24, Fig. 4c–e) is sourced from the ITF thermocline water from south of Java which is then deflected northward by the SETIO eddies (Fig. 3a–c green line, σ_θ_ = 24–26) in the backdrop of a prolonged northwestward flowing SJC (Fig. 4c,e). There is also a secondary ITF freshwater pathway from the Sunda Strait (Figs 4b and 3a–c, green circle-line)^14,56^.

## Itf Fraction in the Indian Ocean

Previous work showed the ITF fraction on σ_θ_ = 24 in the SEC^23^ but lacked information as to how the ITF spreads into the interior of the Indian Ocean. Here, we calculated the ITF water fraction in specific locations of the Indian Ocean using a linear isopycnal mixing method^35^ utilizing the ORAS4 reanalysis and the Argo dataset (2005–2012). The method assumes ITF water at 10.5–15.5°S, 115°E intruding into the Indian Ocean mixed waters. The newly discovered ITF fraction off Sumatra in the mixed layer (σ_θ_ = 23.5) is stronger during the GSWS period (0.24) than in the GSW period (0.035). This result is consistent with the stronger north-westward SJC during the GSWS period that transports the ITF waters from south of Java (Figs 3c, 4d,e, Table S2). This represents a pathway of the ITF that has never been documented before. The ITF fraction in the Mozambique Channel is linked to the Agulhas Current, mixing with the Arabian Sea water and the Red Sea water, and has a higher fraction in the GSWS period (0.093) than in the GSW period (0.085) at the thermocline level (σ_θ_ = 25.5). At intermediate depths (σ_θ_ = 27.4), the IIW fraction is slightly higher in the GSW period (0.738) than in the GSWS period (0.725) (Table S3). The ITF fraction dominates the EGC at thermocline and intermediate depths (Table S4), but there are lower ITF fractions at thermocline and intermediate depths off the Western Australian coast during the GSWS period. The lower salinity at intermediate layers contributes to a slightly lower IIW fraction in the Mozambique Channel and off Western Australia that might be caused by the addition of fresher AAIW from the Southern Ocean (Fig. 2f, Table S4).

## Barrier Layer Thickness (Blt) Changes

**Figure 5:**
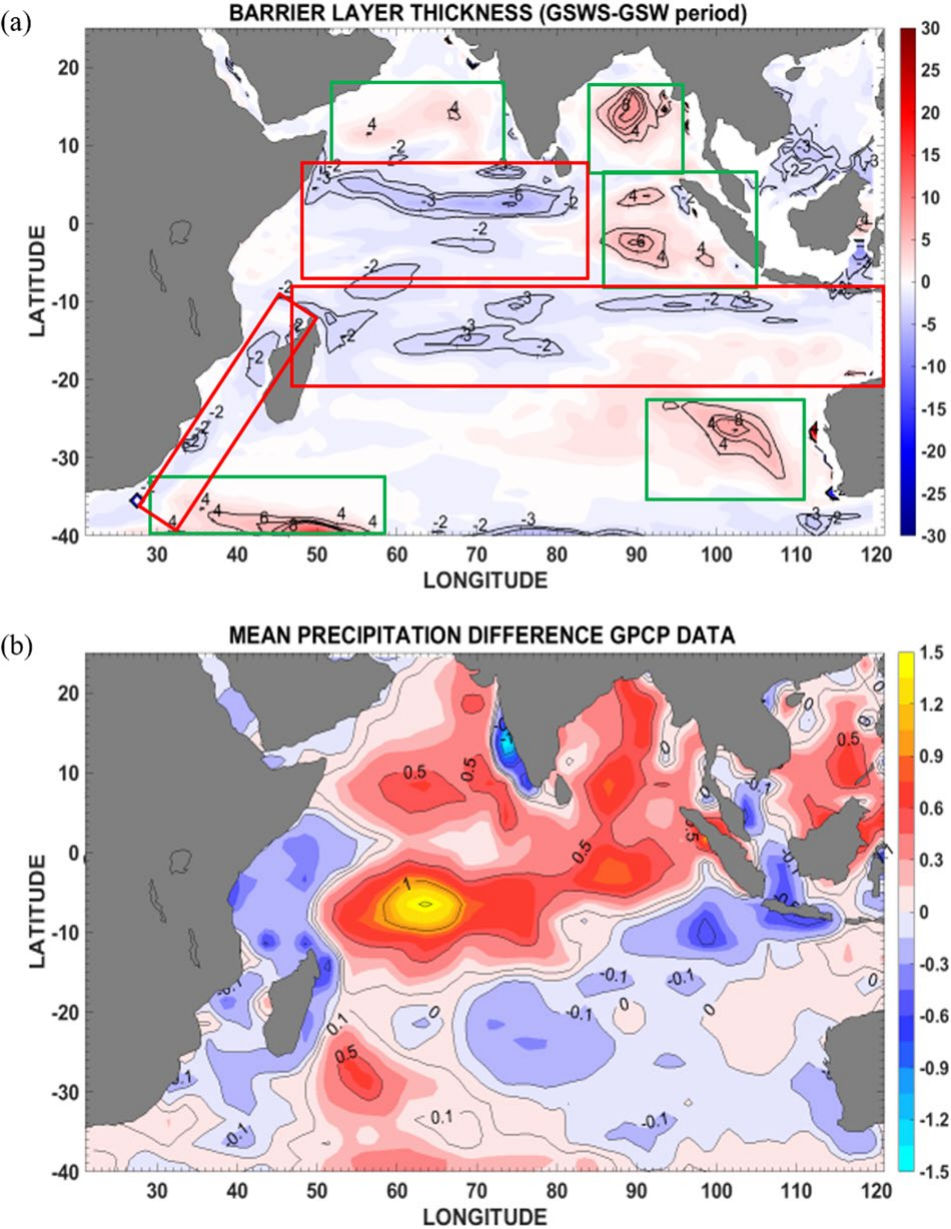
Barrier layer thickness (BLT) and mean precipitation differences in the Indian Ocean. (**a**) BLT difference (unit: meter) = BLT GSWS – BLT GSW period (**b**). Mean precipitation difference (unit: mm/day) = mean precipitation (2000–2012) - mean precipitation (1979–1999). Red box represents the area of reduced BLT and green box the area of enhanced BLT.

The BLT in the SETIO region is an important factor for the Indo-Pacific climate system. Freshwater input from Bay of Bengal^54^ and low salinity ITF water^55^ can change the upper ocean stratification and produce barrier layers. During the GSWS, the IOD-like positive net freshwater flux and the enhanced ITF advection into the ocean both act to impact the upper ocean stratification. Thinner BLT (approx. 2–6 m) generally occurred along the ITF pathways including in the SEC, the Mozambique Channel, and the southwest monsoon drift regions, which can be explained by the reduced rainfall except in the western SEC region (50–80°E) (Fig. 5a, red box). Less freshwater flux into the ocean means that temperature plays a dominant role in shaping the oceanic mixed layer (Fig. 5b) and so reduces the BLT. The more pronounced rainfall over the western SEC region seems due to the enhanced austral summer rainfall along the southwest-northeast tilted inter-tropical convergence zone (ITCZ). This rainfall pattern also corresponds to that found during positive IODs; indeed, positive IODs occurred more frequently in the GSWS period (Figs 5b, S7a,b).

The reduced BLT in the western SEC region (50–80°E) indicates the non-local nature of the upper ocean stratification, which is closely related to the strong ocean dynamics and air-sea interaction over the Seychelle Dome^61^. This region is characterized by rather complex physical processes, such as thermocline dome upwelling, that are unique within the whole tropical Indian Ocean basin. Of interest, this region did not show a direct response to more rainfall during GSWS period. This phenomenon could be due to several possible reasons. First, the thermocline dome upwelling is likely enhanced during GSWS, which is consistent with the increased salinity in this region. Second, such an enhanced upwelling possibly outweighs the rainfall and horizonal advection effects. Third, local air-sea interaction effects could have potentially modulated the impact of the rainfall in the region. All these effects need further exploration.

The reduced BLT in the Mozambique Channel seems to be a direct consequence of the less rainfall during GSWS. However, the reduced BLT within the southwest monsoon drift current region in the southern Arabian Sea may be due to the advection effect from the drier western Indian Ocean. The increased BLT during the GSWS (in the Arabian Sea, the Bay of Bengal, off west Sumatra, and off Western Australia, Fig. 5a, green box), was mainly due to the enhanced rainfall in the two pronounced rain bands lying at 15°N and slightly south of the equator associated with the ITCZ. The rainfall anomaly pattern (Fig. 5b) exemplifies the enhanced South Asian monsoon which is arguably associated with decadal variability^62^. Off West Sumatra and west of Australia, the positive rainfall and intensified ITF transport both help set the larger BLT.

## Summary and Discussion

**Figure 6:**
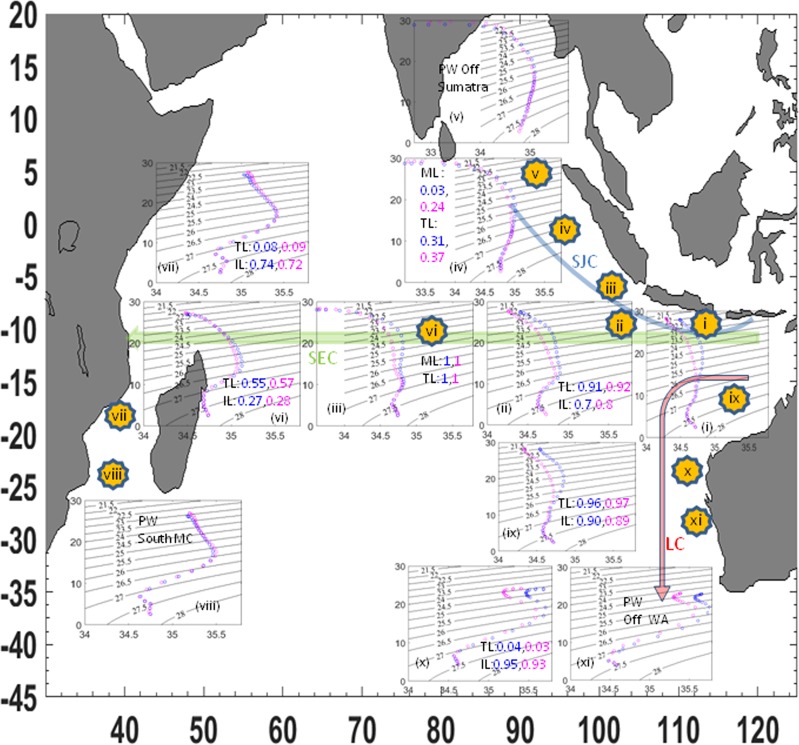
The temperature-salinity (T-S) along ITF pathways, its fate, and the ITF fractions in the Indian Ocean. A new pathway of the ITF water in the Indian Ocean associated with the South Java Current (blue arrow), the ITF water following the South Equatorial Current (SEC, green arrow), the ITF feeding into the Leeuwin Current (LC, red arrow). (i) The T-S as ITF water from the Indonesian exit passages (10–15°S, 115°E), (ii) T-S within the South East Tropical Indian Ocean eddies (10.5–15.5°S, 110.5–114.5°E), (iii) T-S off Sunda Strait (6.5–10.5°S, 100.5–107.5°E), (iv) T-S off Sumatra (0–6.5°S, 95.5–105.5°E), (v) T-S parent water north of Sumatra (0–3.5°N, 95.5–100.5°E), (vi) T-S mid-SEC pathway (10.5–15.5°S, 70.5–90.5°E), (vii) T-S in the mid-Mozambique Channel (15.5–23.5°S, 36.5–45.5°E), (viii) T-S parent water south of Mozambique Channel (22.5°S, 42.5–43.5°E), (ix) T-S ITF water feeding LC within the Eastern Gyral Current (11.5–15.5°S, 115.5–119.5°E), (x) T-S off Western Australian Coast (23–26.5°S, 113°E) and (xi) T-S parent water south off Western Australian Coast (26.5°S, 112.5°E). Each inset shows temperature (°C; y axis) and salinity (psu; x axis), with ML: the mixed layer, TL: the thermocline layer; IL: the intermediate layer and the ITF fractions are listed (blue: the Global Surface Warming period, pink; the Global Surface Warming Slowdown period), (Tables S2–S4). T-S parent water is likely as the fate of ITF water profile within that route.

Pacific waters spreading across the Indian Ocean take various routes from their entry point via the Indonesian archipelago. Here, we highlight the main ITF routes and their fates in the Indian Ocean on decadal time scales (Fig. 6). During GSWS, fresher ITF water that had accumulated within the Indonesian Seas^63^ flowed via a previously unidentified route directly from south of Java to off Sumatra, associated with the stronger SEC that resulted in a prolonged northwestward SJC (Fig. 6). Evidence for the role of the eddies transporting the ITF water to Sumatra is revealed by the shared T-S characteristics within all the ITF profiles of South of Java, the SETIO eddies, and off Sunda Strait (Fig. 6, insets (i–iii)). During the GSWS period, the ITF fraction off Sumatra consisted of 24% of the ITF water from South of Java. Further confirmation might be obtained through eddy-resolving ocean simulations, and this is left for a future study.

The near surface freshwater is a result of mixing between the ITF thermocline water from south of Java with the ITF water exiting via Sunda Strait and the fresher Bay of Bengal water (Fig. 3a–c, 0–3.5°N, 95.5–100.5°E, Fig. 6v). This new ITF pathway may be important for the ITF influence of water mass characteristics associated with intraseasonal upwelling in this region. Ultimately this pathway will also potentially lengthen the passage of ITF waters into the Atlantic Ocean as part of the global thermohaline conveyor. The ITF waters off Sumatra can also be linked directly to the Mozambique Channel via the Rossby waves that are known to influence the Agulhas eddies (Fig. 2e). Our analysis shows that the ITF water reaches the Mozambique Channel after mixing with the Red Sea Water with a direct link to the Agulhas Current from the thermocline down to the intermediate depths (Fig. 3a–c, 22.5°S, 42.5–43.5°E, Fig. 6viii). The ITF waters off the Western Australian coast at intermediate depth mix with the AAIW (Fig. 3a–c, 26.5°S, 112.5°E, Fig. 6xi). During the GSWS period the ITF transport and fraction are stronger in the Indian Ocean, but at intermediate depths the IIW appears inhibited by the stronger AAIW intrusion from the Southern Ocean (Fig. 6, Tables S2–S4). Indeed, the stronger ITF transport contributes to freshening of the Indian Ocean, causing decadal changes in barrier layer thickness that may in turn influence climate variability such as the IOD via air-sea interactions.

The ITF transports nutrients through the exit passages, impacting the biogeochemical cycling in the Indian Ocean^64^ which sustains the health of the marine ecosystem^65^. Thus, long-term changes of the ITF that occurred during GSWS, including the stronger ITF advection in the Southern Indian Ocean that altered the ocean heat content over the upper 700 m^66^, are not only expected to exert an impact on climate but also on fisheries that are important for the regional economy. Our study highlights the importance of monitoring the ITF and its far-reaching pathways within the Indian Ocean, such as the SEC, the Agulhas Current and the Agulhas Leakage, in order to better understand changes in physical and biogeochemical water properties derived from the ITF within the Indian Ocean.

## Method

### The OHC

The primary datasets used in this study are the monthly temperature and salinity of the Ocean Reanalysis System data (ORAS4)^67^. The Ocean Heat Content (OHC) was calculated as a depth-integrated temperature multiplied by the density of water for the entire of the Indian Ocean basin. We use depth values provided by ORAS4 dataset. The OHC anomaly was constructed as the deviation from the mean in the Indian Ocean basin (20.5°N–40.5°S, 20.5–120.5°E). The GSWS Period was defined from 2000 to 2009 and the GSW period from 1958 to 1999.

### Transport variability (ITF, Agulhas Current and Agulhas Leakage)

Total transport for ITF water (10.5–15.5°S, 115.5°E), Agulhas Current (34.5°S, 27.5–30.5°E), and Agulhas Leakage (34.5–36.5°S, 26.5°E) were calculated from the ORAS4 mean zonal or meridional velocity multiplied by delta depth and the transect distance during GSWS and GSW periods. The transport anomaly for each transect was determined from the 1958–2009 mean transport along that transect. To determine whether changes in transport were statistically significant at 99% level we used a *t*-test.

The lead-lag correlations from the time series transport of the ITF water (10.5–15.5°S, 115.5°E), Agulhas Current (34.5°S, 27.5–30.5°E), and Agulhas Leakage (34.5–36.5°S, 26.5°E) are used to estimate the time that ITF water from the Indonesian exit passages may reach the Agulhas Leakage region. In addition, the interannual variability of the ITF geostrophic transport has been examined using the Expendable Bathythermograph (XBT) data which reveals an interplay of ENSO and IOD and exhibits a strengthening trend linked to enhanced Pacific Trade winds during the hiatus period^68^. The XBT data are assimilated in the ORAS4 reanalysis system^67^. Figure S8 shows a snapshot of annual evolution of temperature profile during 2008 near the ITF exit that, as expected, is comparable between the two datasets albeit the ORAS4 is somewhat smoother.

### Diagnosing ITF spreading along isopycnal

The salinity range from the ITF profiles in the Indonesian Sea exit passages (34.5, 34.6 and 34.8 psu) adopted from (Fig. 2 of ref.^20^) on σ_θ_ (23.5, 25.5 and 27.41) represented the ITF water in the mixed layer, thermocline layer and intermediate layer, respectively. Salinity contours were calculated by averaging salinity values from the ORAS4 dataset. Delta Salinity was calculated as the Salinity during the GSWS period minus the Salinity in the GSW period. Similar methods were also applied to the Argo (2005–2012) dataset^69^ (Fig. S1).

### ITF water subduction and SJC northward deflection

To investigate the ITF thermocline layer off Sumatra that was sourced from the northwestward SJC, we used Temperature, Salinity and Velocity from the ORAS4 dataset averaged on σ_θ_ of 23.5, 24, and 24.5 for the GSWS and GSW periods for seasonal mean periods March-April-May (MAM) and, June-July-August (JJA). To investigate whether a decadal time scale of SJC deflection off Sumatra has different patterns in the GSWS and GSW periods we used Delta Velocity (averaged velocity GSWS period - averaged velocity GSW period) and Delta Salinity (averaged salinity GSWS period - averaged salinity GSW period). We found the prolonged SJC northwestward deflection toward Sumatra in MAM as well as in JJA during the GSWS period. The Monsoon Onset Monitoring and Its Social Ecological Impacts (MOMSEI) CTD cruise confirmed the ITF mixed layer presence off Sumatra (Fig. 4c, track BCD) on intra-seasonal time scales, while off Sunda Strait the ITF was captured from the surface to 500 m depth (Fig. 4c, track A). This provides evidence that the ITF from Sunda Strait entering into the Indian Ocean and the ITF thermocline water from south of Java occupied the site off Sumatra. The ERA 20 C wind dataset^70^ shows the enhanced easterly wind generated during the prolonged SJC of the GSWS period.

### T-S diagram analysis using ORAS4 and Argo dataset

The T-S diagrams in the boxes within the Indian Ocean (Fig. 3) were calculated by averaging the salinity and temperature from the Argo dataset over the period 2005–2012 and from the ORAS4 dataset over the period 1958–2009. The density values were calculated from each dataset using the equation of state of seawater.

### Determining ITF fraction in the Indian Ocean

We assumed that the ITF water (10.5–15.5°S, 115°E) is an intrusion of water into the Indian Ocean that spreads along isopycnals (σ_θ_ 23.5, 25.5 and 27.4) by linear mixing via three routes: the route off Sumatra uses the T-S profile north of Sumatra (0–3.5°N, 95.5–100.5°E) as the parent water; the route following the SEC uses the profile south of Mozambique Channel (22.5°S, 42.5–43.5°E) as the parent water; and the route via the Western Australian coast uses the profile (26.5°S, 112°E) as the parent water. The parent waters are chosen as the last profile at the end of ITF routes that still indicate the presence of thermocline ITF water (IUW)/IIW content, likely as the fate of ITF water profile within that route. We adopt a conservative along-isopycnal mixing law of salinity (equation 2 in ref.^35^) to calculate the ITF fractions for every T-S profile along each route of the ITF pathway.

$$ITF\,fraction\,at\,a\,given\,location=\,(\frac{Salinity\,location-Salinity\,parent\,water}{Salinity\,ITF\,{10.5}^{\circ }-{15.5}^{\circ }{\rm{S}},\,{115}^{\circ }{\rm{E}}-Salinity\,parent\,water})$$

The ITF fractions were calculated for T-S profile at locations: off Java-Sunda Strait (6.5–10.5°S, 100.5–107.5°E), off Sumatra (0–6.5°S, 95.5–105.5°E) for the ITF water deflection to off Sumatra; SETIO eddies (10.5–15.5°S, 110.5–114.5°E), mid-SEC pathway (10.5–15.5°S, 70.5–90.5°E), mid-Mozambique Channel (19.5°S, 42.5–43.5°E) for the ITF following SEC; and the profile EGC area (11.5–19.5°S, 115.5–119.5°E) and off Western Australian coast (22.5–25.5°S, 113°E) (see Tables S2–S4). The fraction of parent water in a given location is given by (1-ITF fraction). The ITF water fraction values are calculated for specific locations in the Indian Ocean (see Tables S2–S4), and the fate of ITF waters are described from the parent water profiles (Fig. S3a–c).

### Barrier layer thickness analysis and precipitation pattern in the Indian Ocean

The stronger advection of ITF water during the GSWS period affects the upper layer salinity stratification in the Indian Ocean, inducing changes in mixed layer depth and so affecting the thicknesss of the barrier layer. Here the isothermal layer depth is the layer where the temperature is 0.5 °C lower than SST and the mixed layer depth is the layer where the density is 0.125 kg/m^3^ higher than at the surface^71^ (Fig. S5a–f). Barrier Layer Thickness (BLT) is defined by the difference between isothermal layer depth and the mixed layer depth (Fig. S6a,b). Note the BLT mean values during GSWS period are in agreement with a previous study^72^ that used a different data set. The Global Precipitation Climatology Project (1979–2015)^73^ (GPCP) data was used to determine annual mean precipitation patterns in the Indian Ocean during the GSW and GSWS periods (Fig. S6c,d).

The mean SST difference (unit: degree Celsius) = mean SST (2000–2009) – mean SST (1958–1999), and the time series of Dipole Mode Index (DMI) for the time period of 1958–2009 were used to diagnose the BLT pattern during the GSWS period and provided support that the precipitation patterns were similar to those found during IODs.

The Dipole Mode Index (DMI) for time period of 1958–2015 is available at: https://stateoftheocean.osmc.noaa.gov/sur/ind/dmi.php.

### Watermass list

**Table:** 

**No**	**Watermasses**	**Temperature Range**	**Salinity Range**	**σ**_**θ**_	**Reference**
1	BBW (Bay of Bengal Water)	25°–29 °C	28–35 psu	21–23	ref.^46^
2	IUW (Indonesian Upper Water)	8°–23 °C	34.4–35 psu	23–27	ref.^46^
3	IEW (Indian Equatorial Water)	8°–23 °C	34.6–35 psu	23.5–27	ref.^46^
4	SICW (South Indian Central Water)	8°–25 °C	34.6–35.8 psu	23.5–27	ref.^46^
5	ASW (Arabian Sea Water)	24°–30 °C	35.5–36.8 psu	24–25	refs^46,47^
6	PGW (Persian Gulf Water)	5°–14 °C	34.8–35.4 psu	26–27	refs^46,47^
7	RSW (Red Sea Water)	5°–14 °C	34.8–35.4 psu	27–27.5	refs^46,47^
8.	IIW (Indonesian Intermediate Water)	3.5°–5.5 °C	34.6–34.7 psu	27–27.5	ref.^46^
				27.25–27.52 (18°S)	ref.^25^
9.	AAIW (Antartic Intermediate Water)	2°–10 °C	33.8–34.8 psu	26.5–27.5	ref.^46^
				27.1–27.3	ref.^47^
				27.2–27.6 (10° N, 17°S, 32°S, 43°S)	ref.^44^
				27.2–27.4 (20°S)	ref.^25^

## Supplementary information

SUPPLEMENTARY INFORMATION
