# Supplementary material for: Previously unidentified Indonesian Throughflow pathways and freshening in the Indian Ocean during recent decades
Source: Sci Rep. 2019 May 14;9:7364. doi: 10.1038/s41598-019-43841-z (PMC6517581; doi:10.1038/s41598-019-43841-z)
Supplement: Supplementary file 1 — SUPPLEMENTARY INFORMATION [file 41598_2019_43841_MOESM1_ESM.docx]

**SUPPLEMENTARY INFORMATION**

**Previously unidentified Indonesian Throughflow pathways and freshening in the Indian Ocean during recent decades**

Salvienty Makarim^1,2^*, Janet Sprintall^3^, Zhiyu Liu^1^, Weidong Yu^4^, Agus Santoso^5,6^_,_  Xiao-Hai Yan^1,7^, and R. Dwi Susanto^8,9^

^1^State Key Laboratory of Marine Environmental Science, and Department of Physical Oceanography, College of Ocean and Earth Sciences, Xiamen University, Xiamen, China

^2^Agency for Marine and Fisheries Research and Development, Ministry of Marine Affairs and Fisheries, Jakarta, Indonesia

^3^Scripps Institution of Oceanography, University of California San Diego, La Jolla, CA, USA

^4^First Institute of Oceanography, SOA, Qingdao, China

^5^Climate Change Research Centre and ARC Centre of Excellence for Climate Extremes, University of New South Wales, Sydney, Australia

^6^Centre for Southern Hemisphere Oceans Research, CSIRO Oceans and Atmosphere, Hobart, Australia

^7^College of Earth, Ocean and Environment, University of Delaware, Newark, DE, USA

^8^Department of Atmospheric and Oceanic Science, University of Maryland, College Park, MD, USA

^9^Faculty of Earth Sciences and Technology, Bandung Institute of Technology, Bandung, Indonesia

Corresponding author: Salvienty Makarim ([smakarim2013@stu.xmu.edu.cn](mailto:smakarim2013@stu.xmu.edu.cn), [s3lvi2009m@gmail.com](mailto:s3lvi2009m@gmail.com)).

**
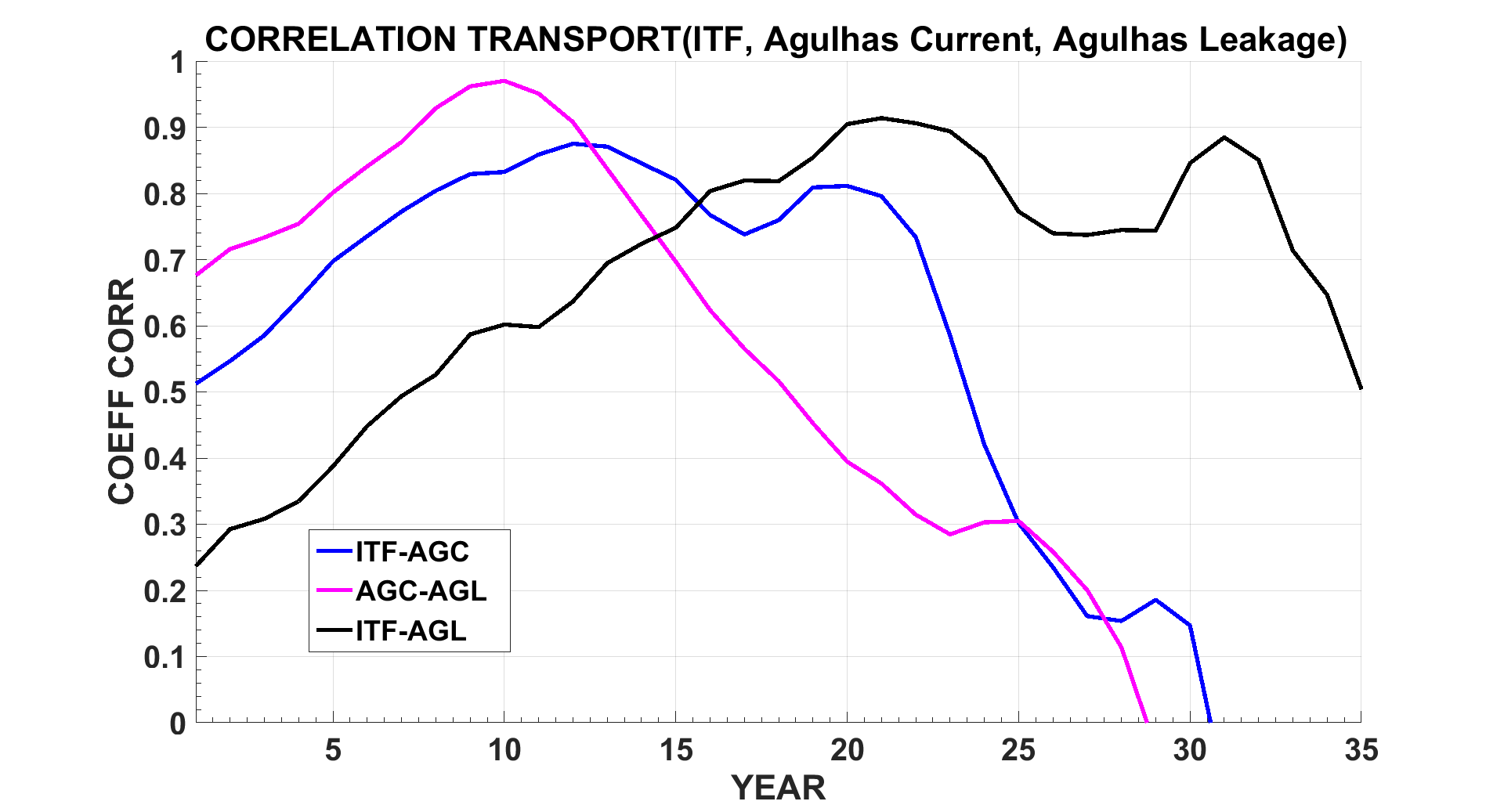
**

**Figure S1. Lead-lag correlation for the interannual transports (1958-2009) in Fig 1c.**  The ITF pathways (10.5-15.5° S, 115.5° E), the Agulhas Current (34.5° S, 27.5-30.5° E), and the Agulhas Leakage (34.5-36.5° S, 26.5° E). Maximum correlations occur when the ITF water from the Indonesian exit passages leads the Agulhas Current by 12 years (blue line); the Agulhas Current leads the Agulhas Leakage by 10 years (pink line); and the ITF water from the Indonesian exit passages leads the Agulhas Leakage by around 20 years (black line).

| 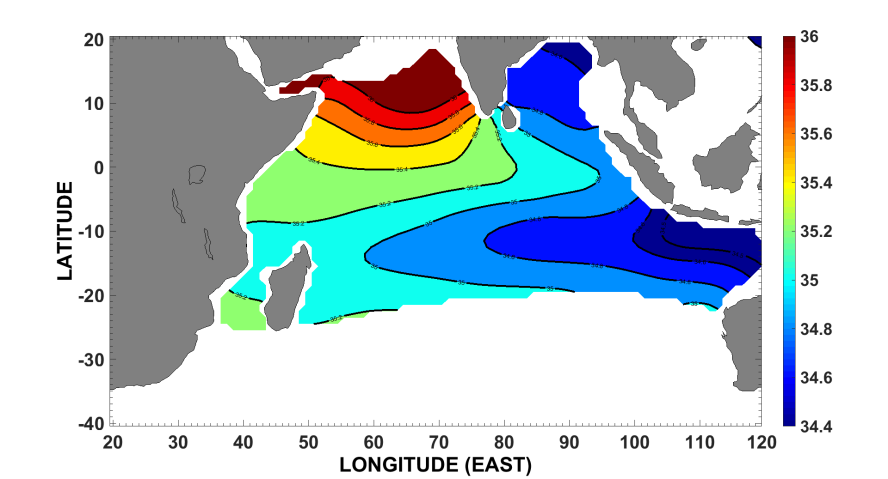  (a) |
| --- |
| 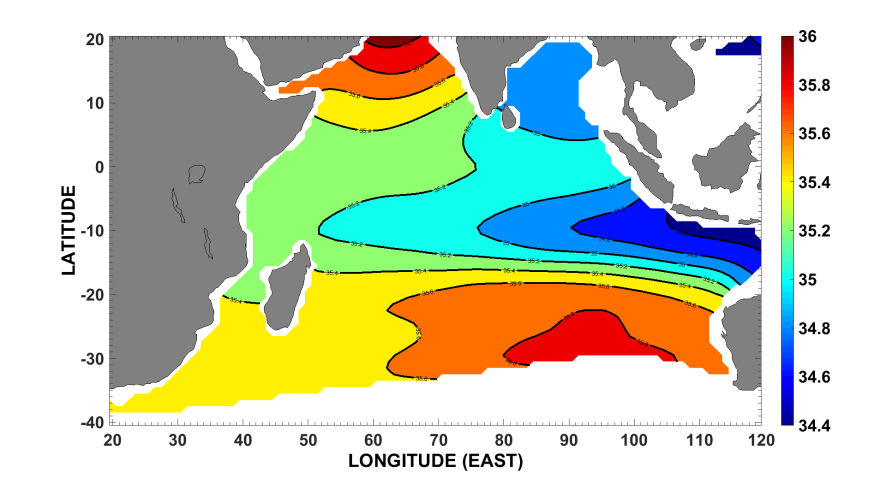  (b) |
| 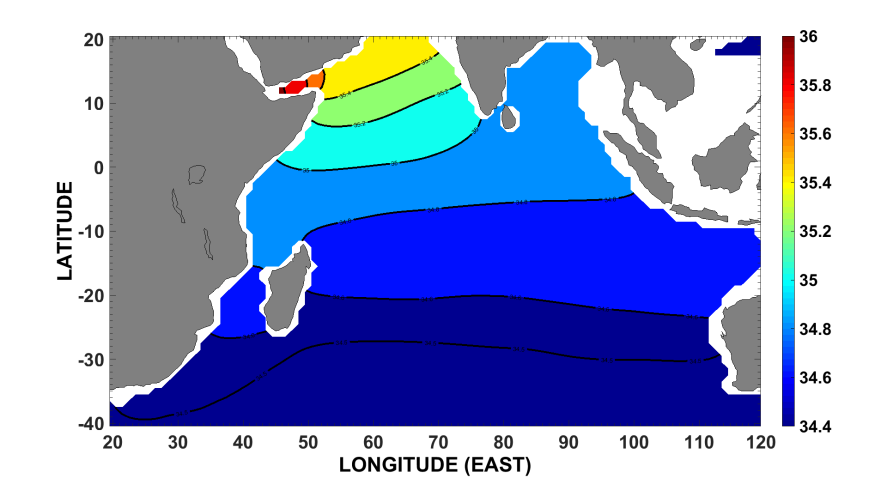  (c) |

**Figure S2.** **ITF spreading in the Indian Ocean during Global Surface Warming Slowdown (GSWS) Periods using Argo data. a**. Isopycnal contour for σ_23.5_ from Argo dataset (2005–2010). Yellow box area (off Sumatra) indicates where the Argo data are insufficient so the ORAS4 data are used. MOMSEI cruise data within this region also confirmed the water mass was within the ITF salinity range. **b**. Isopycnal contour for σ_25.5_ from the Argo dataset (2005–2010). Red box area indicates where the Argo data are insufficient so the ORAS4 data are used. **c**. Isopycnal contour σ_27.41_ sigma from Argo dataset (2005–2010).

| 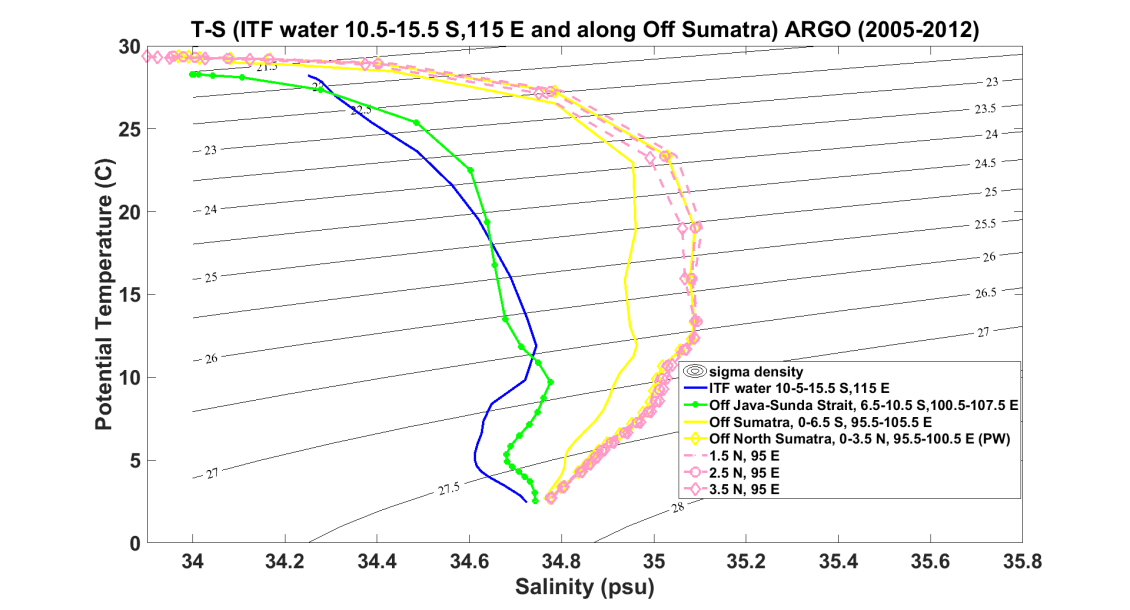  (b)  (a) |
| --- |
| 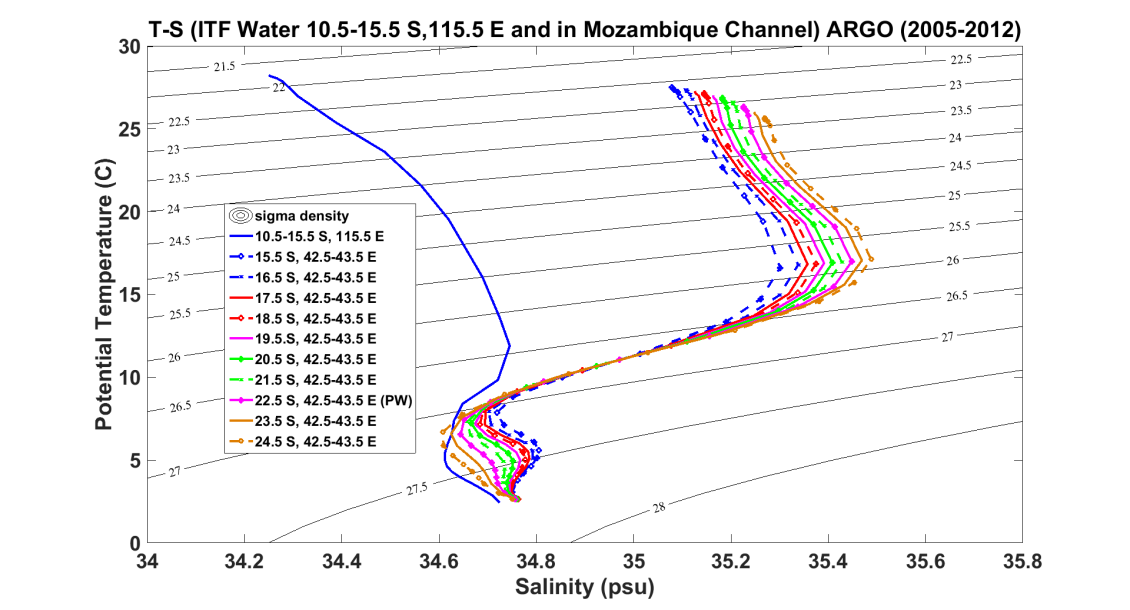  (c) |
| 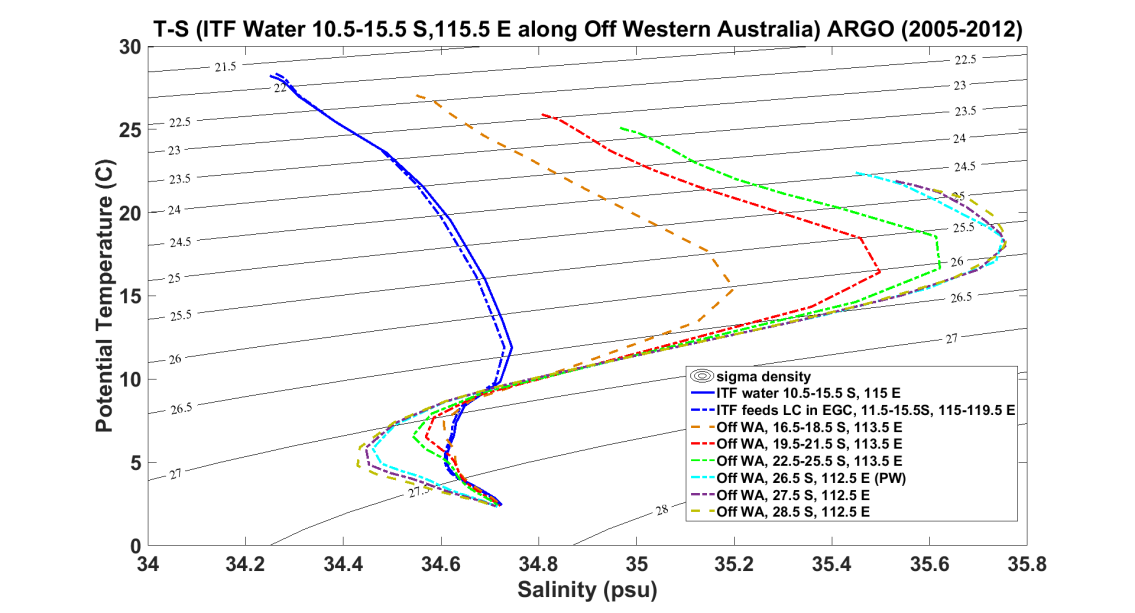 |

**Figure S3. T-S diagram showing the water masses from the ITF source.** a. T-S for the ITF water (10.5-15.5° S, 115° E) and off Sumatra**.** Purple box represents the ITF water at mixed layer depths and pink box the mixture of ITF water and the Bay of Bengal Water (BBW). The blue arrow indicates the parent water as the latest profile of ITF water mixing with BBW. b. T-S for the ITF water (10.5-15.5° S, 115° E) and in the Mozambique Channel. Green box represents the Indonesian Intermediate Water (IIW) and brown box the change to a salinity maximum induced by the Red Sea Water (RSW). The blue arrow indicates the parent water as the latest profile of IIW mixing with RSW. c. T-S for the ITF water (10.5-15.5° S, 115° E) and along the Western Australian coast. Green box represents the IIW, black box the Antarctic Intermediate Water (AAIW) and red box the mixture of IIW and AAIW. The blue arrow indicates the parent water as the latest profile of IIW mixing with AAIW.

| 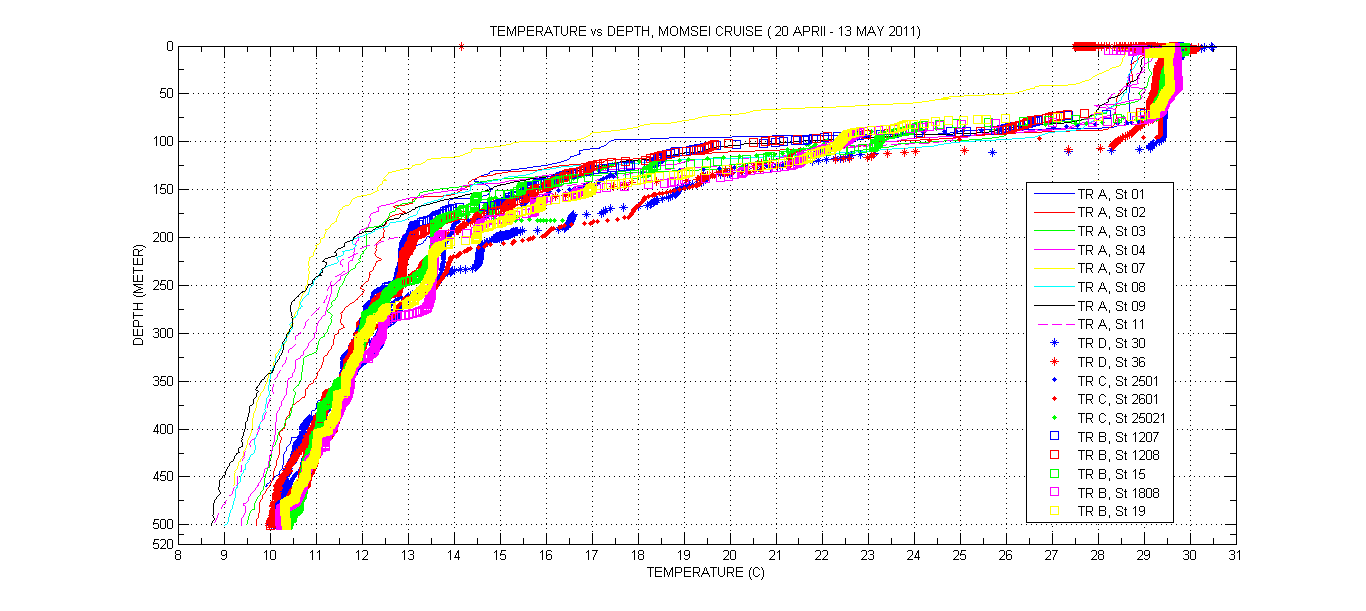  (a) |
| --- |
| 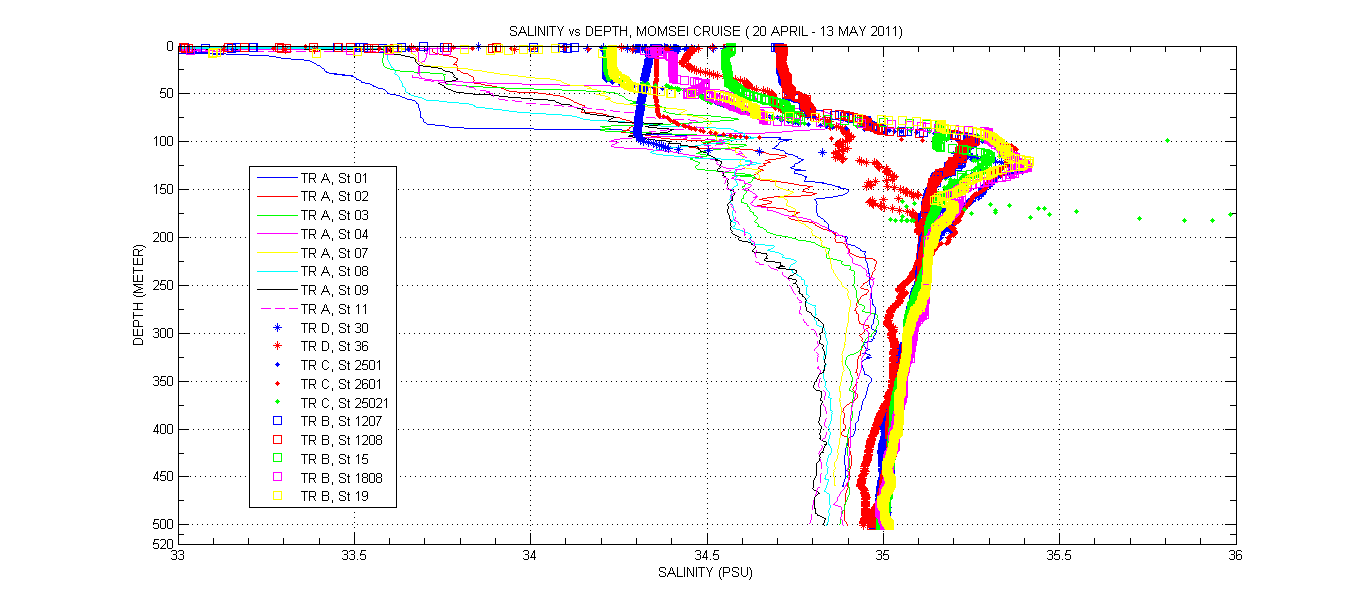  (b) |

**Figure S4. Temperature and Salinity profiles from CTDs collected during the MOMSEI Cruise (20 April**–**13 May 2011). a.** Temperature vs Depth, and **b**, Salinity vs Depth. The different colours and symbols represent the profile for each CTD station. Red box indicates the mixed layer depth of the ITF water off Sumatra (70-90 m depths) in the salinity range (34.5-34.8 psu) and in the temperature range (26-29° C).

| **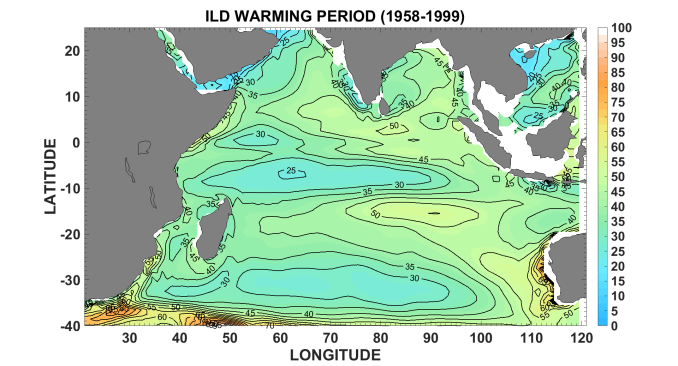**  (a) | **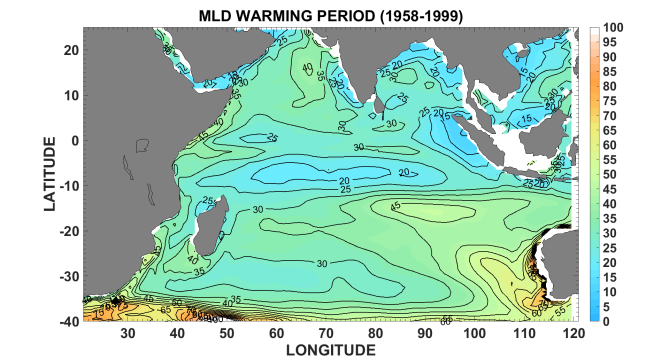**  (d)b |
| --- | --- |
| **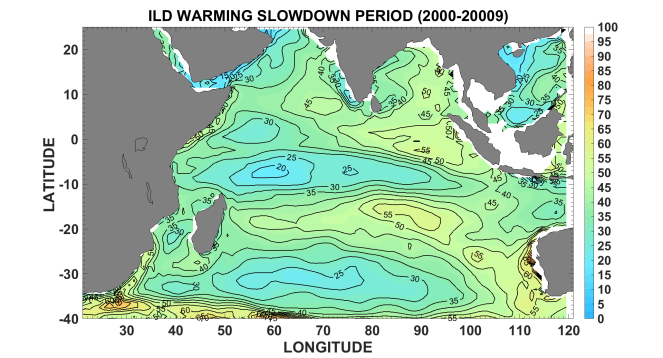**  (b) | **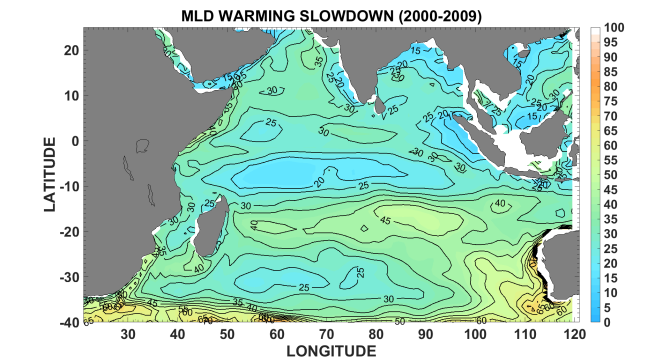**  (e) |
| **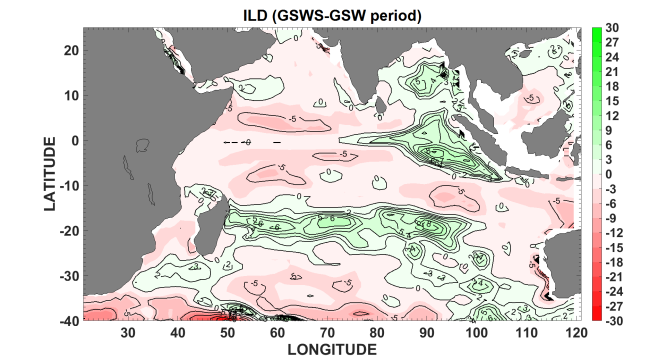**  (c) | **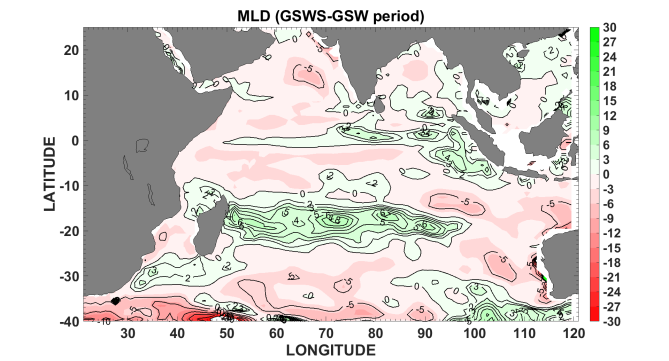**  (f) |

**Figure S5. Isothermal Layer and Mixed layer Depths.** a, Isothermal layer during the GSW (1958–1999), b. Isothermal layer during the GSWS (2000–2009), c. Delta Isothermal Layer, d. Mixed layer in the GSW (1958–1999), e. Mixed layer in the GSWS (2000–2009), f. Delta Mixed Layer.

| 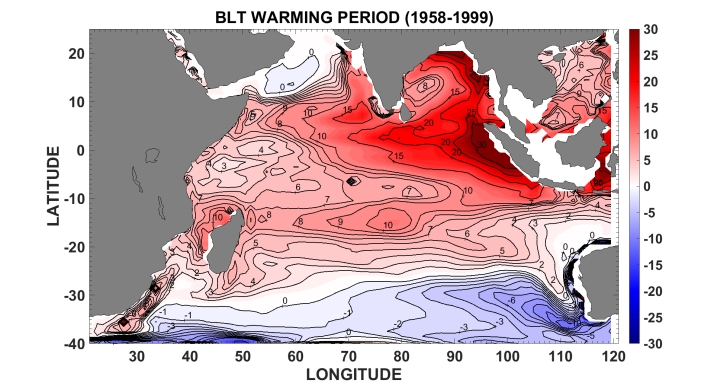  (d)  (b)  (a) | 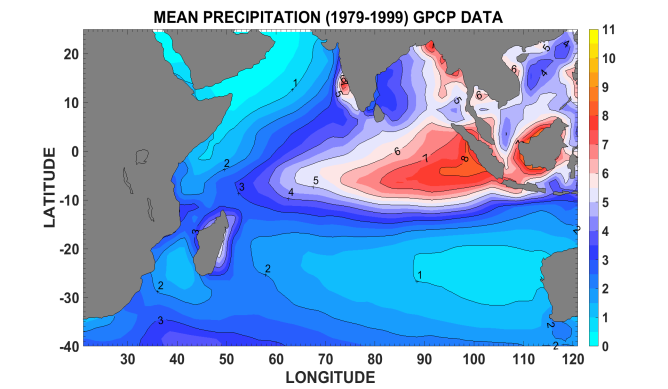  (c) |
| --- | --- |
| 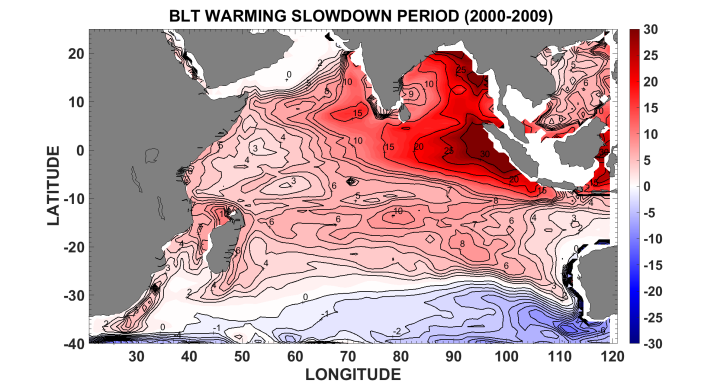 | 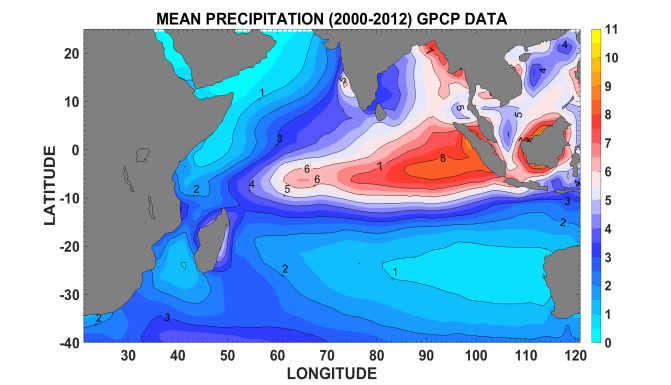 |

**Figure S6. Barrier layer thickness (BLT) and precipitation patterns in the Indian Ocean. a,** BLT in the GSW period. **b,** BLT in the GSWS period. **c**, Mean precipitation in 1979–1999, **d** Mean precipitation in 2000–2012.

| 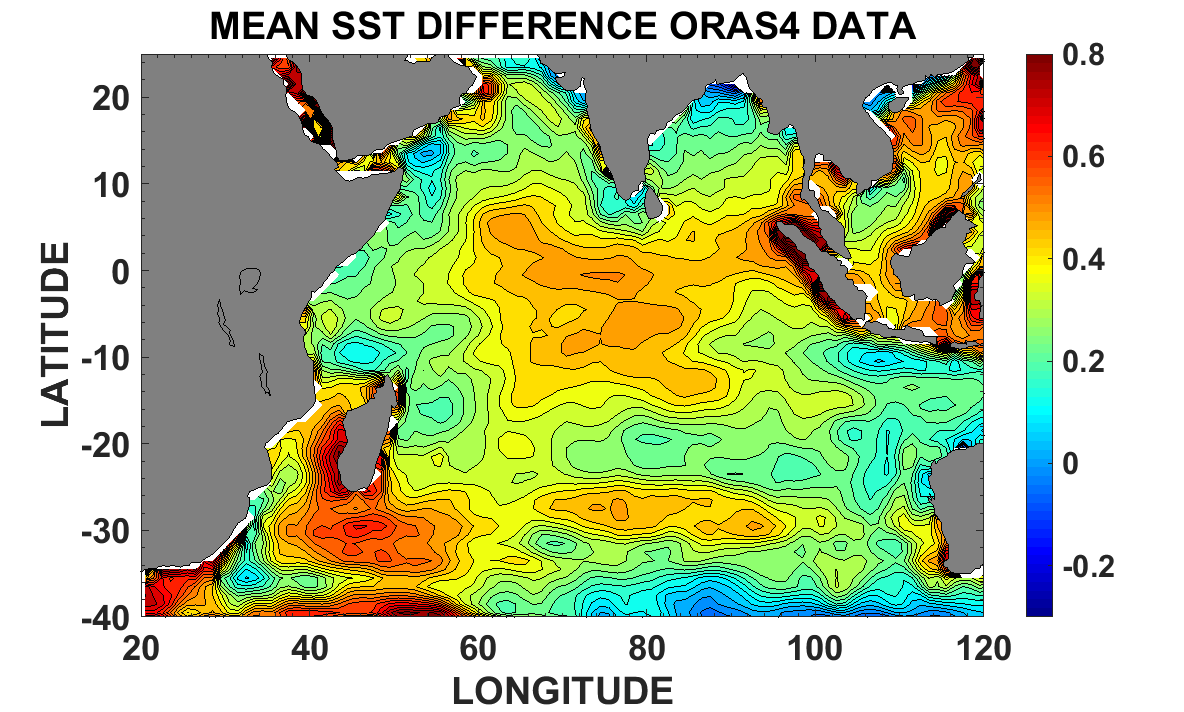  (a)  (b) |
| --- |
| 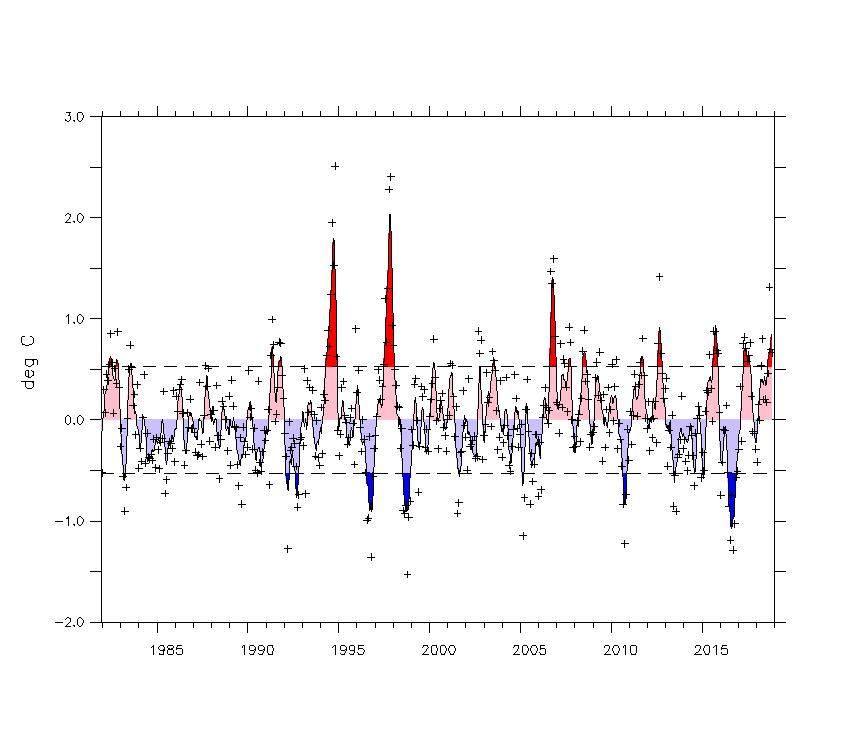 |
|  |

**Figure S7. The mean sea surface temperature (SST) from ORAS4 data and the Dipole Mode Index (DMI). a**, The mean SST difference (unit: degree Celsius) = mean SST (2000-2009) – mean SST (1958-1999). **b**, The DMI for the period 1958-2015, available at https://stateoftheocean.osmc.noaa.gov/sur/ind/dmi.php.

The mean SST differences are consistent with the more frequent positives IODs that occurred during the GSWS period.


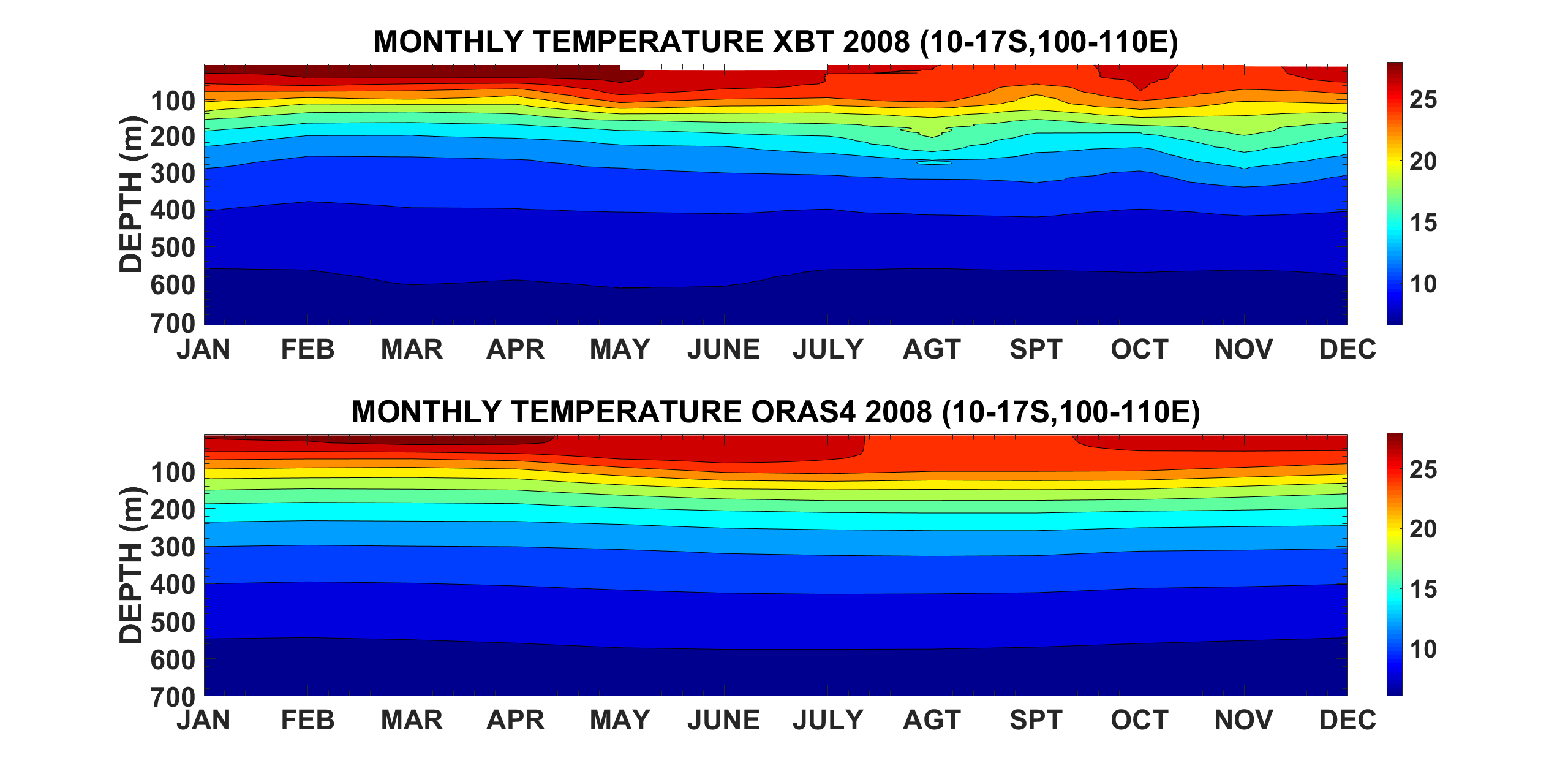


**Figure S8. The monthly-mean temperature in 2008 near where the ITF exits from the Indonesian Seas into the Indian Ocean (10-17 S, 100-110 E).** The data are based on **a,** Expendable Bathythermograph (XBT), and **b,** ORAS4**.** Both data sets show a similar pattern in the temperature profiles although, as expected, the ORAS4 is somewhat smoother. This suggests that ORAS4 can be used to trace the ITF within the Indian Ocean.

| **Region** | **Transport in GSW (Sv)** | **Transport in GSWS (Sv)** |
| --- | --- | --- |
| ITF water (10.5-15.5° S, 115.5° E) | 8.46 | 8.8 |
| Agulhas Current (34.5° S, 27.5-30.5° E) | -39.05 | -40.18 |
| Agulhas Current Leakage (34.5-36.5° S, 26.5° E) | -37.09 | -40.29 |

**Table S1. ITF water, Agulhas Current and Agulhas Current Leakage transport in the GSW and GSWS periods.** Total transport for ITF water (10.5-15.5° S, 115.5° E), Agulhas Current (34.5° S, 27.5-30.5° E), and Agulhas Leakage (34.5-36.5° S, 26.5° E) during the GSWS period were stronger than during the GSW period. These results show a stronger ITF transport will enhance transport of the Agulhas Current and the Agulhas Leakage. This suggests that the water leakage from the Indian Ocean will increase into the south Atlantic Ocean.

| ITF Northward Deflection (SJC) | σ_23.5_ | | | σ_25.5_ | | |
| --- | --- | --- | --- | --- | --- | --- |
|  | ORAS4  GSW | ORAS4  GSWS | Argo | ORAS4  GSW | ORAS4  GSWS | Argo |
| Off Sunda Strait (6.5-10.5° S, 100.5-107.5° E) | 1 | 1 | 0.861 | 1 | 1. | 1 |
| Off Sumatra (0-6.5° S, 95.5-105.5° E) | 0.035 | 0.240 | 0.150 | 0.315 | 0.374 | 0.351 |

**Table S2.** The ITF fraction associated with the South Java Current (SJC) flow to Sumatra.

| ITF follows SEC pathways | σ_23.5_ | | | σ_25.5_ | | | σ_27.4_ | | |
| --- | --- | --- | --- | --- | --- | --- | --- | --- | --- |
|  | ORAS4  GSW | ORAS4  GSWS | Argo | ORAS4  GSW | ORAS4  GSWS | Argo | ORAS4  GSW | ORAS4  GSWS | Argo |
| SETIO eddies (10.5-15.5° S, 110.5-114.5° E) | 1 | 0.978 | 0.923 | 0.915 | 0.92 | 0.853 | 0.707 | 0.802 | 0.537 |
| Mid SEC pathway (10.5-15.5° S, 70.5-90.5° E) | 0.845 | 0.64 | 0.603 | 0.55 | 0.569 | 0.476 | 0.273 | 0.282 | 0.629 |
| Mozambique Channel(15.5-23.5°S, 36.5-45.5° E) | 0.237 | 0.149 | 0.066 | 0.085 | 0.093 | 0.065 | 0.738 | 0.725 | 1 |

**Table S3.** The ITF fraction within the main South Equatorial Current (SEC) pathway.

| ITF associated with Leeuwin Current | Sigma 25.0 | | | Sigma 27.25 | | |
| --- | --- | --- | --- | --- | --- | --- |
|  | ORAS4  GSW | ORAS4  GSWS | Argo | ORAS4  GSW | ORAS4  GSWS | Argo |
| LC in EGC (11.5-15.5° S ,115.5-119.5° E) | 0.969 | 0.974 | 1 | 0.906 | 0.896 | 0.962 |
| Off Western Australia (23-26.5° S, 113° E) | 0.041 | 0.038 | 0.202 | 0.959 | 0.939 | 0.821 |

**Table S4.** The ITF fraction associated with the Leeuwin Current.
